# Xenobiotics and Broiler Microbiota: Molecular Insights into Bacterial Antimicrobial Resistance and Food Safety Implications for Human Health

**DOI:** 10.3390/jox15040129

**Published:** 2025-08-08

**Authors:** Marta Gonçalves, Nuno Vale, Paulo Martins da Costa, Paula Silva

**Affiliations:** 1 School of Medicine and Biomedical Sciences (ICBAS), University of Porto, Rua de Jorge Viterbo Ferreira, 228, 4050-313 Porto, Portugal; mcgoncalves@icbas.up.pt (M.G.); pmcosta@icbas.up.pt (P.M.d.C.); 2 PerMed Research Group, RISE-Health, Faculty of Medicine, University of Porto, Alameda Professor Hernâni Monteiro, 4200-319 Porto, Portugal; nunovale@med.up.pt; 3 RISE-Health, Department of Community Medicine, Health Information and Decision (MEDCIDS), Faculty of Medicine, University of Porto, Alameda Professor Hernâni Monteiro, 4200-319 Porto, Portugal; 4 Laboratory of Personalized Medicine, Department of Community Medicine, Health Information and Decision (MEDCIDS), Faculty of Medicine, University of Porto, Rua Doutor Plácido da Costa, 4200-450 Porto, Portugal; 5 Interdisciplinary Centre of Marine and Environmental Research (CIIMAR), Terminal de Cruzeiros do Porto, de Leixões, Av. General Norton de Matos s/n, 4450-208 Matosinhos, Portugal; 6 Laboratory of Histology and Embryology, Department of Microscopy, School of Medicine and Biomedical Sciences (ICBAS), University of Porto, Rua Jorge Viterbo Ferreira 228, 4050-313 Porto, Portugal; 7 iNOVA Media Lab, ICNOVA-NOVA Institute of Communication, NOVA School of Social Sciences and Humanities, Universidade NOVA de Lisboa, 1069-061 Lisbon, Portugal

**Keywords:** poultry, antibiotics, antimicrobial resistance, One Health

## Abstract

Antibiotics have played an evolving role in poultry production, generally transitioning from widespread use to more precise and controlled applications. Despite this shift, the long-term consequences of earlier practices continue to affect current and future generations. This review aims to explore the multifaceted consequences of antibiotic use in poultry production, with particular emphasis on the growing challenge of antimicrobial resistance (AMR). Evidence demonstrates that antibiotic use affects the gut microbiome, often resulting in long-lasting decreased diversity and restructuring of the bacterial communities. Moreover, consequences extend to the surrounding environment, including the airborne microbiome, water systems, and poultry litter, where alterations in microbial communities tend to be more subtle, whereas changes in genetic elements related to resistance are often more pronounced (drift). The emergence and persistence of resistance in these environments facilitate the spread of resistance genes across ecological boundaries, contributing to the broader dissemination of AMR. These findings highlight the complex, interconnected nature of AMR, underscoring the urgent need for responses grounded in the One Health framework. Such approaches are essential for safeguarding both public and environmental health while maintaining sustainable poultry production practices.

## 1. Introduction

Antibiotics have been extensively used in broiler production for several years, with poultry production representing the leading branch of livestock production. Nevertheless, in Europe, there has been a remarkable decline, driven by both regulatory frameworks and the industry’s efforts to appeal to and impress consumers [1,2]. These compounds are some of the most common xenobiotics introduced into animal husbandry [3,4]. As biologically active compounds that are foreign to both host organisms and their microbial ecosystems, antibiotics exert considerable selective pressure on microbial populations [5]. Throughout the following sections, the term antibiotic will be used broadly to refer to all antimicrobial agents. However, antibiotics are natural compounds produced by microorganisms that inhibit the growth of other microorganisms. These compounds are commonly used for therapeutic and prophylactic purposes in broiler systems. While growth promotion persists in some regions, it is highly restricted and banned in the European Union [6,7]. Commonly used classes include tetracyclines (e.g., chlortetracycline and doxycycline), sulfonamides, macrolides, fluoroquinolones (e.g., enrofloxacin), and beta-lactams [8]. Once introduced into the broiler gut or farm environment, antibiotics can alter microbial composition and function. They are primarily employed for their ability to selectively eliminate pathogenic microorganisms, thereby facilitating the re-establishment of a balanced microbial ecosystem (eubiosis or normobiosis). Nonetheless, their use is inherently associated with adverse effects, including the perturbation of commensal microbiota (dysbiosis), facilitation of the emergence of antibiotic-resistant bacteria, and promotion of the dissemination of antimicrobial resistance genes (ARGs) in both pathogenic and commensal microorganisms. These effects extend beyond the gastrointestinal tract; antibiotic residues and resistant bacteria are excreted in feces, accumulate in litter, and become airborne within poultry houses, further expanding their ecological footprint [9,10,11]. As persistent xenobiotics, many antibiotics, particularly tetracyclines and fluoroquinolones, can persist in manure, soil, and water long after administration, thereby posing risks to agroecosystems and public health. Their presence in animal-derived food products and environmental compartments supports the spread of resistance determinants across microbial communities and between animal and human hosts [12]. This narrative review seeks to provide a critical analysis of the available evidence on the role of antibiotics as xenobiotics in poultry production, with the aim of elucidating the invisible pathways of human exposure, the ecological mechanisms underlying the spread of antimicrobial resistance, and the public health implications, through the lens of the One Health approach and the ethical imperative of precaution.

## 2. Microbial Reservoirs of Antimicrobial Resistance in Broiler Production Environments

### 2.1. The Role of Antibiotics in the Broiler Industry and Their Broader Implications

The discovery of antibiotics represents a pivotal milestone in human civilization. Although this breakthrough is usually attributed to Doctor Alexander Fleming, for his discovery of penicillin, microorganisms have used these compounds for several millennia. Therefore, the natural patent for antibiotics belongs to the microorganisms themselves, which use these molecules to compete and survive [13]. In this review, the term xenobiotics refers to biologically active substances, including antibiotics of natural, semi-synthetic, and synthetic origin, introduced into the host or environment where they are foreign and exert selective ecological pressure, consistent with definitions used in environmental and food safety sciences [14,15,16].

Antibiotics are frequently defined as substances that inhibit microorganisms at clinical concentrations. However, it is essential to understand that the effects of antibiotics vary greatly depending on their concentration. Therefore, at high concentrations above the Minimal Inhibitory Concentration (MIC), they act as lethal toxins, resulting in bacterial death. The key nuance pertains to the observation that at intermediate concentrations, antibiotics function as stress inducers, thereby blocking cellular division and promoting DNA mutations, genetic recombination, and eventually DNA repair, which leads to the appearance of acquired resistance. Finally, at lower concentrations, antibiotics act as signaling molecules that modulate gene expression, not through mutations, but often result in antibiotic tolerance and, consequently, survival [17].

These behaviors become even more evident when the environmental impact is considered. A substantial proportion of antibiotics is excreted into the environment, generating a concentration gradient between the administration site and the ecosystem; this gradient creates sub-inhibitory conditions that favor the emergence of resistance [18].

The role of antibiotics in animal production extends beyond that of therapeutic agents. In fact, these xenobiotics have been employed for metaphylaxis, which consists of treating and preventing cases of disease in the group, and prophylaxis, with the aim of preventing the overall appearance of the disease [19]. Moreover, in 1940, it was discovered that animals fed *Streptomyces* mycelium residues grew faster. Initially, researchers thought that the growth resulted from the protein content of the micelles. However, they quickly realized that antibiotics play an important role in this growth [20]. Many justifications for the role of antibiotics as growth promoters include their ability to eliminate subclinical infections, improve nutrient absorption, and decrease microorganism abundance, leading to decreased competition for nutrients and reduced production of inhibitory metabolites [21]. The emergence of resistance due to the overuse of antibiotics has led to legislation across several regions. In 2019, the European Union significantly restricted the use of antibiotics in livestock, banning routine and prophylactic group treatments with antimicrobials, limiting metaphylactic use to high-risk situations, and prohibiting prophylactic usage in medicated feed to promote animal welfare and reduce environmental impacts [22,23].

### 2.2. Antibiotics as Xenobiotics: Environmental and Health Perspectives

Antibiotics utilized in poultry production, regardless of their natural, semi-synthetic, or fully synthetic origin, act as xenobiotics in environmental and host ecosystems. The term xenobiotic, traditionally defined as a substance foreign to a biological system, extends beyond its classical application to purely synthetic chemicals, and is extensively employed in environmental and toxicological sciences to denote any biologically active compounds introduced into organisms or ecosystems [14,15]. Although many antibiotics used in poultry, such as tetracyclines and macrolides, are derived from microbial sources, they remain exogenous to poultry and the surrounding environment, where they exert selective pressure on microbial communities, persist as residues, and contribute to the dissemination of resistance genes [16]. Notably, the high concentrations at which these antibiotics are administered far exceed the trace levels at which they function as natural microbial signaling molecules, transforming them into strong ecological drivers of mutation and horizontal gene transfer [24]. In addition, their persistence in environmental matrices facilitates the recruitment of previously unrecognized resistance genes and disrupts the natural microbial equilibria [25]. Table 1 provides a summary of the primary antibiotic classes used in poultry production, their origins, and the rationale for classifying them as xenobiotics. This clarification aligns with our broader environmental and One Health perspective, which considers the ecological impact of all antimicrobial agents, irrespective of their synthetic or natural origin.

### 2.3. Fecal Microbiota and the Gut Resistome

In addition to their antimicrobial activity, antibiotics act as xenobiotics in broiler chickens, disrupting the ecological balance of the gut microbiota and altering host–microbe interactions. The gut microbiome is a dynamic ecosystem that is essential for nutrient metabolism, immune modulation, and pathogen resistance. Antibiotic administration alters its composition, diversity, and functional capacity, with consequences extending well beyond its primary antimicrobial effects [28].

Numerous studies have shown that antibiotic exposure induces treatment- and age-dependent shifts in gut microbial communities. For example, bacitracin supplementation has been associated with increased *Enterococcaceae* and reduced *Rikenellaceae* in broilers raised under high stocking densities, without affecting *alpha* diversity (diversity within a sample) but producing clear changes in *beta* diversity (diversity between samples) profiles [29]. Enrofloxacin reduced *Bacteroidetes* and increased *Firmicutes* while depleting other genera, such as *Anaerotruncus*, *Dorea*, *Lactobacillus*, and *Oscillospira* [30]. These findings illustrate the selective pressure exerted by antibiotics, which promotes the expansion of some taxa while suppressing others.

Although several studies have not demonstrated a clear link between antibiotic administration and reduced microbial diversity, this relationship is well established in both human and veterinary medicine [31,32]. Recent studies have highlighted similar trends in broiler production [33]. Hence, the effect of antibiotics on microbial diversity is context-specific and depends on the compound used. Some agents reduce richness and evenness, especially shortly after treatment [30,34], whereas others, such as chlortetracycline, may paradoxically increase diversity indices [35]. This is particularly relevant in the context of broiler production when the impact of alpha diversity on performance is considered, as higher diversity is normally associated with higher performance and, consequently, a lower feed conversion ratio (FCR) [36]. Remarkably, as birds mature, microbial communities tend to converge across treatment groups, with host age becoming a stronger determinant of microbiota structure than antibiotic exposure [37]. This suggests a degree of resilience or restoration of capacity following xenobiotic-induced perturbations.

**Figure 1:**
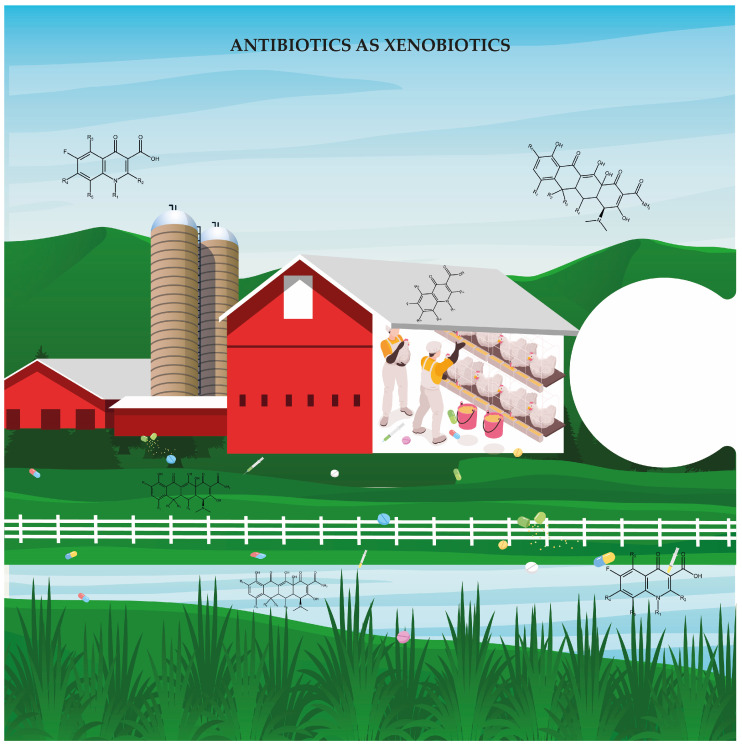
Antibiotics as xenobiotics. This is the first piece of a complex puzzle that must be considered when addressing antibiotic resistance. In poultry farms, antibiotics act as xenobiotics and foreign chemical substances introduced into the environment. They accumulate in soil and water, disrupt microbial ecosystems, and create selective pressures that favor the emergence and persistence of antimicrobial resistance.

Functional alterations in the microbiome further underscore the systemic effects of antibiotics such as xenobiotics (Figure 1). Antibiotic growth promoters (AGPs) such as narasin and virginiamycin have been shown to enhance pathways related to nitrogen metabolism, urea degradation, and heme biosynthesis while diminishing mucin polysaccharide use [34]. Such shifts may contribute to improved growth performance but may also modify the host–microbiome metabolic interface in unpredictable ways. In contrast, enrofloxacin has been associated with changes in amino acid, fatty acid, and sugar metabolism and disruptions in mTOR signaling, a key regulator of cellular growth [30]. These observations highlight how antibiotics can reprogram not only microbial composition but also metabolic output.

The most concerning consequence of antibiotic exposure is the selection and further enrichment of ARGs, which reinforces the classification of antibiotics as xenobiotics. ARG profiles change significantly following exposure to AGPs, with resistance emerging not only to the administered drugs but also to unrelated classes. For instance, virginiamycin and bacitracin increase the abundance of genes conferring resistance to beta-lactams and macrolide–lincosamide–streptogramin B antibiotics, despite no structural relationship with these compounds [35]. Enrofloxacin has been shown to increase plasmid-mediated quinolone resistance genes, such as *qnrS*, potentially owing to horizontal gene transfer (HGT) within the gut microbiome [30]. HGT is often mediated by mobile genetic elements (MGEs) such as integrons (int1 and int2) and transposases (tnpA, IS613) [38,39]. Integrons are mobile genetic elements that capture and express resistance gene cassettes under the control of a single promoter. This coordinated expression allows for a simple genetic event to confer resistance to multiple antibiotic classes, thereby amplifying the spread of multidrug resistance across microbial communities [40].

**Figure 2:**
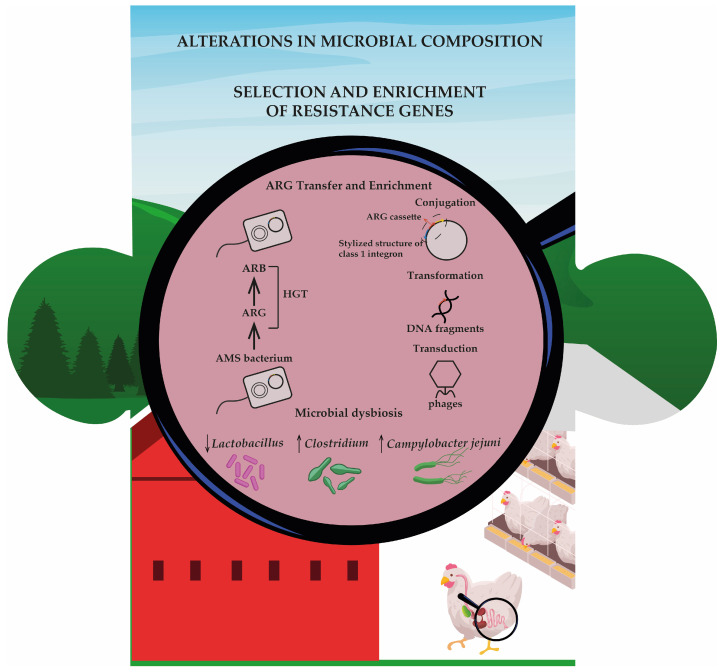
This second piece of the puzzle illustrates how antibiotic use in poultry farming promotes both antimicrobial resistance and alterations in gut microbiota. In the gut of broiler chickens, antibiotic pressure results in antimicrobial-resistant bacteria (ARB) and drives the horizontal gene transfer (HGT) of antibiotic resistance genes (ARGs) through conjugation, transformation, and transduction. Simultaneously, microbial dysbiosis occurs, characterized by a decline in beneficial genera (*Lactobacillus*) and proliferation of opportunistic or pathogenic taxa (*Clostridium*, *Campylobacter jejuni*). AMS: antimicrobial-sensitive bacteria.

The persistence and mobility of ARGs further highlights the xenobiotic footprint of antibiotics. The resistance of genes not only increases during treatment but can persist across production cycles. Genes such as *bla*_TEM_ (beta-lactamase) and *qnrB* (quinolone resistance) may increase in copy number over time [41]. Functional metagenomics has revealed novel resistance genes with limited similarity to known ARGs, thus revealing an underexplored reservoir of resistance within poultry microbiomes [41]. The possibility that these genes could be transferred to zoonotic pathogens or enter the human microbiota underscores their broader ecological and public health implications (Figure 2).

In addition to resistance genes, antibiotic exposure plays a key role in shaping the composition and function of the gut microbiome in poultry. The selective pressure exerted by antibiotics can also lead to the persistent enrichment of resistant taxa. For example, *Campylobacter* levels are higher in birds treated with bacitracin or bacitracin methylene disalicylate, raising concerns regarding food safety [29,37]. The depletion of protective taxa, such as *Lactobacillus*, during antibiotic treatment may facilitate colonization by opportunistic pathogens. Although *Campylobacter* increases naturally with age, antibiotic-treated birds often carry higher loads than control birds, potentially requiring stricter biosecurity or processing interventions [37].

The ecological impact of antibiotics is shaped by their spectrum of activity and responsiveness to the microbial communities. Some AGPs, such as bacitracin, may enrich beneficial taxa, such as *Lachnospiraceae*, without affecting the overall diversity [35], whereas others, including lincomycin and tylosin, reduce butyrate-producing taxa, such as *Faecalibacterium* and *Lactobacillus*, which are vital for gut barrier function and host immunity. Although certain microbiome shifts have been linked to improved performance via reduced microbial competition, increased metabolic versatility, or enhanced nutrient availability [34], the long-term implications of these shifts must be considered alongside the risks of dysbiosis and resistance.

In conclusion, antibiotics used in broiler production act as xenobiotics with far-reaching consequences. They disrupt the microbial community structure, modulate metabolic functions, and promote the selection of ARGs, with significant implications for animal health, productivity, and food safety. Although some functional outcomes may be beneficial in production contexts, the growing recognition of resistance dissemination and microbiome disruption highlights the need for more prudent antibiotic use and alternative strategies. Framing antibiotics as xenobiotics provides a valuable lens through which their ecological and evolutionary effects on host-associated microbial ecosystems can be evaluated.

### 2.4. Airborne Microbial Communities in Poultry Houses

Antibiotic administration during broiler production significantly alters the composition and resistance profiles of airborne microbial communities in poultry houses. These enclosed systems contain complex bioaerosols composed of environmental bacteria and resistant strains originating from feces, feathers, dust, and feed [42]. Antibiotic use, particularly via in-feed formulations, modifies both the microbial structure and the prevalence of ARGs in air samples, posing risks to both animal and human health [43,44].

Airborne microbial populations in broiler environments are dominated by members of the phyla *Firmicutes*, *Actinobacteria*, *Bacteroidetes*, and *Proteobacteria*, with *Staphylococcus* and *Bacillus* being frequently detected as the most prevalent genera [45,46]. Antibiotic pressure, particularly from tetracyclines, macrolides, and aminoglycosides, drives the selection of resistant strains within these taxa. For instance, *Staphylococcus aureus* isolates recovered from bioaerosols exhibit multidrug resistance, including β-lactams and macrolides, with methicillin-resistant *S. aureus* (MRSA) strains identified in both air and fecal samples [44,47]. Remarkably, MRSA harboring SCC*mec* types I and IV, commonly associated with hospital- and community-acquired infections, has been recovered from poultry environments, highlighting the potential for zoonotic transmission [47].

In addition to resistant pathogens, poultry house air harbors a broad array of ARGs, including those conferring resistance to tetracyclines (*tetM*, *tetC*, and *tetO*), sulphonamides (*sul1* and *sul2*), macrolides (*ermB* and *ermC*), quinolones (*qnrA* and *qnrS*), and aminoglycosides (*strA* and *strB*) [38,43,48]. Among these, *tetM* and *sul1* frequently emerge as dominant and can reach concentrations higher than those reported in hospital air samples [49]. These findings reflect the strong selective pressure within broiler environments and the high potential for aerosolized resistance dissemination.

Environmental conditions, particularly seasonality, also shape the dynamics associated with HGT, and consequently, ARG dissemination in confined poultry housing. During winter, reduced ventilation and increased particulate matter, especially fine particles (PM2.5), contribute to higher airborne ARG loads and favor HGT transmission pathways [48,49].

Multiple studies have identified fecal matter as the primary source of airborne ARGs in poultry facilities. The compositional similarities between cloacal and air samples support the hypothesis that antibiotic exposure in the gut leads to the shedding of resistant bacteria, which are subsequently aerosolized [38,39]. Ventilation systems and air currents may facilitate the dispersion of ARGs within and beyond the farm environment [42,44].

Efforts to mitigate airborne microbial loads via disinfection present complex challenges. Spray disinfectants, such as hypochlorous acid, glutaraldehyde–decamethonium bromide, and sodium dichloroisocyanurate, are commonly used but have shown limited effectiveness under field conditions. In some cases, their use has paradoxically increased microbial diversity and the relative abundance of ARGs, potentially owing to sublethal selective pressures or the disruption of microbial equilibrium [50]. These findings question the reliability of current sanitation protocols for controlling airborne antimicrobial-resistant microorganisms (AMR).

The implications for occupational and public health are significant. Poultry workers are at an increased risk of respiratory exposure to ARG-laden aerosols, which may lead to colonization or infection by multidrug-resistant organisms [44]. Genes such as *tetM* and *mecA* have been detected in air and dust samples, indicating potential routes for their integration into the human microbiome [44,47]. Moreover, aerosolized ARGs can extend beyond poultry house boundaries and contaminate adjacent soils, crops, and atmospheric environments [38,39].

Seasonal factors can amplify this risk. During winter, the contribution of fecal matter to airborne bacterial loads increased from approximately 20% in the summer to nearly 60%, accompanied by an increase in the likelihood of HGT events to as high as 78%. [48]. Comparative studies have shown that farms using in-feed antibiotics exhibit markedly higher airborne ARG concentrations. For example, *tetM* gene abundance in air samples can reach nearly 7.5 log copies/m^3^ in medicated chickens, whereas it remains negligible in antibiotic-free systems [43]. These results clearly demonstrate the role of antibiotics in shaping airborne resistomes. In summary, extensive evidence supports a strong and multifactorial link between antibiotic use in broiler production and the structure and resistome of airborne microbial communities in poultry farms. This relationship is influenced by antibiotic administration, microbial ecology, environmental conditions, and sanitation protocols. Although the presence of resistant bacteria in poultry bioaerosols is well established, key knowledge gaps remain, particularly concerning long-term exposure effects, interspecies gene transfer, and effective mitigation strategies. Addressing these challenges will require integrated One Health approaches to reduce AMR risks and to protect both animal and human health.

### 2.5. Drinking Water as Vectors of Resistance

Drinking water systems in broiler farms are increasingly being recognized as reservoirs and transmission routes for antibiotic-resistant bacteria and ARGs. When inadequately maintained, these systems provide favorable conditions for bacterial proliferation, biofilm formation, and genetic exchange, contributing to the persistence and dissemination of resistance across the farm environment. In particular, high microbial loads and diverse ARG profiles have been detected in poultry drinking water, even on farms that do not administer antibiotics [51,52].

Genes conferring resistance to critically important antimicrobials, including *bla*_NDM_ (carbapenems), *bla*_CMY-2_ (cephalosporins), *mcr-3*, *mcr-5* (polymyxins), and *qnrS* (fluoroquinolones), have been identified in aquatic systems [51]. The localization of certain ARGs, such as *mcr-5*, at the distal ends of drinking water pipelines highlights the structural vulnerabilities of these systems [51].

Biofilms play a central role in the maintenance and spread of drug-resistant bacteria. Low flow rates, nutrient accumulation from medicated treatments, and ambient temperatures within water distribution systems foster biofilm development [53,54]. These biofilms provide a protective matrix that supports dense bacterial communities, shields them from disinfection, and facilitates HGT. Opportunistic and pathogenic taxa, such as *Pseudomonas* spp., *Stenotrophomonas* spp., and extended-spectrum β-lactamase (ESBL)-producing *Escherichia coli*, thrive within these niches [52,55]. Even on organic farms, ARGs such as *ermB*, *bla*_TEM-1_, and *bla*_CTX-M-1_-like remain prevalent, suggesting that environmental dissemination, rather than pharmacological pressure, maintains these resistance genes [51].

Microbial composition and ARG abundance varied across matrices. Fecal samples tend to harbor a broader range and higher concentrations of ARGs, such as *oqxA* and *bla*_SHV_, likely because of gut-specific dynamics and vertical transmission from breeder flocks [51]. In contrast, genes such as *bla*_NDM_ and *mcr-5* were more frequently found in water and biofilm samples, indicating that these environments serve as complementary resistance reservoirs. Biofilm communities differ significantly from planktonic populations, with families such as *Sphingomonadaceae* and *Comamonadaceae* playing key roles in ARG persistence [51].

Contamination is not limited to industrial farms. In small-scale operations, untreated surface water sources are major entry points for resistant bacteria. Sharmila et al. (2024) reported increasing levels of ESBL-producing *E. coli* in water samples across the production cycle, surpassing the levels in feed or fecal samples by day 40 [55]. Factors such as fecal shedding, drinker placement on the floor, and lack of regular cleaning are likely to contribute to this cumulative contamination [55].

Antibiotic administration through drinking water can further exacerbate this problem. In South Korea, ciprofloxacin medication via waterlines results in significantly higher levels of high-level ciprofloxacin-resistant *Enterococcus faecalis*. Resistance was also detected in farms that did not use ciprofloxacin, indicating environmental persistence and indirect gene transfer through plasmids via dust or feces [56]. Even after waterline treatment, multidrug-resistant strains, such as *Pseudomonas* spp. and *Ochrobactrum* spp., persist within biofilms, as reported by Mustedanagic et al. (2023) [52]. These communities may also support the survival of co-resident pathogens such as *Campylobacter* spp. [52].

Standard disinfection practices often fail to eliminate biofilm. The physical complexity of pipelines and insufficient contact time limit their efficacy [54]. Although extended disinfectant exposure and mechanical cleaning can reduce microbial load, complete biofilm removal remains elusive. Overaggressive disinfection may also disrupt the beneficial microbiota, raising concerns about unintended consequences for animal health [51].

Emerging evidence from outside poultry houses supports these findings. In Central China, Rui and Qiu (2024) identified ARGs, including *van*T (vancomycin resistance gene), *adeF* (efflux pump gene primarily found in *Acinetobacter baumannii*), and *sul1* (sulfunamide resistance gene), in farm water and nearby reservoirs [57]. Despite policy measures to restrict antibiotic use, these genes persist, reflecting the limits of pharmacological stewardship alone [57]. Some ARGs found in the environment mirror those detected in clinical pathogens, underscoring the One Health relevance of environmental reservoirs as potential sources of antimicrobial resistance transmissible across humans, animals, and ecosystems [25,57].

The persistence of ARGs in water systems, even in the absence of antibiotic input, suggests that mitigation strategies must go beyond drug regulation. These may include improved water system design to reduce stagnation, biofilm formation, more effective biofilm-targeting treatments, and routine surveillance using tools such as metagenomics [57]. A One Health framework that integrates animal, human, and environmental health is essential for the development of comprehensive interventions [51,52].

In conclusion, drinking water in broiler farms functions as both a reservoir and transmission route for antimicrobial resistance. Biofilm resilience, structural limitations of water systems, and microbial diversity contribute to the persistence and dissemination of ARGs. Although fecal matter remains a well-documented source of resistance, the role of drinking water, especially in sustaining resistance in antibiotic-free settings, warrants equal attention. Robust multidisciplinary strategies are urgently needed to disrupt these environmental reservoirs and reduce the risks to animal and public health.

### 2.6. Poultry Litter Microbiome and Its Role in Antimicrobial Resistance Dynamics

Poultry litter (PL), a complex amalgamation of bedding material, feces, feathers, feed residues, and microorganisms, functions as both a valuable agricultural resource and potential reservoir for AMR bacteria and genes. There is increasing global concern regarding the role of PL in the persistence and dissemination of antimicrobial resistance, particularly in high-density broiler production systems. The aim of this subsection is to consolidate recent findings on the composition and dynamics of the PL resistome, the influence of antibiotics, gene transfer mechanisms, environmental impacts, and implications for public and animal health.

Numerous studies have confirmed that PL harbors a vast and diverse resistome, including ARGs against tetracyclines (*tetA–E*), sulfonamides (*sul1*, *sul2*), aminoglycosides (*aadA1*, *aadA2*), macrolides (*ermB*, *ermC*), β-lactams (*bla*_CTX-M_, *bla*_TEM_, bla_CMY-2_), quinolones (*qnrS*, *aac(6′)-Ib-cr*), and colistin (*mcr-1*) [58,59,60,61]. Litter frequently contains multidrug-resistant *E. coli* and *Salmonella* spp., some of which exhibit resistance to as many as 9 of the 11 antibiotics tested [61]. Gupta et al. (2021) observed that vancomycin-resistant *Enterococcus* and fluoroquinolone-resistant *Campylobacter jejuni* persisted in litter throughout the production cycle, indicating that PL serves as a long-term environmental reservoir for clinically significant resistant organisms [62].

The application of antimicrobials exerts a selection pressure that influences both the composition and functionality of the PL resistome. Residual concentrations of antibiotics, including enrofloxacin, ciprofloxacin, bacitracin, tetracyclines, and sulfonamides, are frequently detected in PL, with persistence sometimes exceeding 100 days [63,64]. These residues facilitate the selection and maintenance of ARGs after cessation of active treatment. Gupta et al. (2021) demonstrated that bacitracin increased the prevalence of vancomycin resistance genes and class 2 integrons in litter, while enrofloxacin elevated multidrug-resistant *Enterobacteriaceae* [62]. However, this relationship is complex; the presence of ARGs, such as *qnrS* or *bla*_CTX-M_, in litter does not consistently reflect the resistome of the broiler gut, underscoring the distinct ecological role of litter [62]. Gupta et al. (2021) also indicated that environmental factors and age were more significant determinants of microbiome and resistome variation (cloaca vs. litter) than antibiotic treatment alone, suggesting that selective pressure interacts with microbial succession over time [62].

The reuse of litter, often driven by economic considerations, modifies microbial composition and promotes the accumulation of ARGs. Accumulated litter supports resilient biofilm-forming bacteria and shifts towards taxa adapted to stress and antimicrobial exposure [58,62]. Oxendine et al. (2023) observed that reused litter facilitated the persistence of donor *E. coli* strains with varying plasmid-carrying capacities, underscoring the potential for sustained ARG maintenance [65]. Although reuse prolongs ARG exposure, temporal analyses by Gupta et al. (2021) demonstrated an increase in bacterial diversity throughout the production cycle [62]. This increasing complexity may dilute or amplify resistance, contingent upon prevailing ecological interactions. HGT is generally presumed to occur in PL, owing to the presence of MGEs, integrons (intI1 and int2), and plasmid-encoded ARGs [58,66]. Tripathi et al. (2023) identified shared ARGs and bacterial hosts among litter, soil, and plants—including *ErmF*, *lnuB*, and *bla*_TEM-98_—supporting plasmid-mediated HGT across environmental compartments [66]. However, Oxendine et al. (2023) contested the notion that litter is a highly permissive hotspot of HGT [65]. Under various experimental conditions (including different temperatures, donor/recipient ratios, and media), no transfer of resistance was detected from PL bacteria to a susceptible *Salmonella* strain. The transfer was successful only under controlled in vitro conditions at 25 °C, using specific donor strains and plasmids. These findings suggest that the actual transfer of resistance genes in litter is constrained by multiple environmental and biological barriers [65].

Land application of raw PL constitutes the principal mechanism by which AMR is disseminated from broiler farms to broader environments. Numerous studies have demonstrated that litter introduces ARGs, such as *tetW*, *ermB*, *bla*_CTX-M-32_, and *sul1/2*, into soils, where these genes may persist for extended periods, ranging from weeks to months [59,60,66]. ARGs have been detected in leachates [67] and plant tissues [66], indicating their transfer across multiple trophic levels. Ngogang et al. (2020) associated such applications with increased risks of foodborne outbreaks, noting that *E. coli* and *Salmonella* from untreated litter can survive for months in soil and water [66]. These concerns were corroborated by Agga et al. (2024), who observed that PL-amended soils temporarily harbored ESBL-producing *E. coli*, including *bla*_CTX-M_-positive strains [59].Proper treatment with PL prior to land application significantly mitigated microbial and chemical hazards [59]. Okada et al. (2024) compared composting methods and found that mechanical aeration without co-substrates effectively eliminated *E. coli*, reduced antibiotic residues (e.g., maduramicin), and decreased parasite load [68]. Notably, this method avoids heavy metal accumulation observed in carbon-supplemented composting. Despite these benefits, the adoption of composting remains limited [68]. Barrero et al. (2025) emphasized that even after composting, ARGs can persist unless temperature, pH, and moisture thresholds are rigorously maintained [69]. The presence of ARGs in litter does not always translate into transfer risk, nor does their detection in crops guarantee their pathogenicity. The resistome is not monolithic; its composition and transferability depend on environmental context, selective pressure, and microbial compatibility [62,65]. Despite the overwhelming evidence of the risk, regulatory systems often overlook non-edible matrices such as PL. Ngogang et al. (2020) called for AMR surveillance in chicken litter, especially in low-resource settings, where regulation and treatment are minimal [61]. The finding of 100% multidrug resistance (MDR) among *E. coli* isolates and the use of critically important antibiotics highlight the urgent need for One Health-informed governance. PL is a complex biological system that plays a central role in maintaining and disseminating antimicrobial resistance. It harbors diverse ARGs, supports the survival of MDR pathogens, and serves as a vector for the transfer of resistance genes under specific and restrictive conditions. Land application of untreated litter contributes to ARG enrichment in soils, crops, and water bodies. However, reuse and improper management can increase these risk factors. Using composting to reduce antibiotic use and surveilling high-risk ARGs and pathogens must be prioritized. Managing PL within a One Health framework is not only prudent but also essential for limiting the environmental amplification of AMR [61].

## 3. Antibiotics as Xenobiotics: Invisible Pathways to Human Exposure

### 3.1. Fragmented Exposure and Hidden Burdens

**Figure 3:**
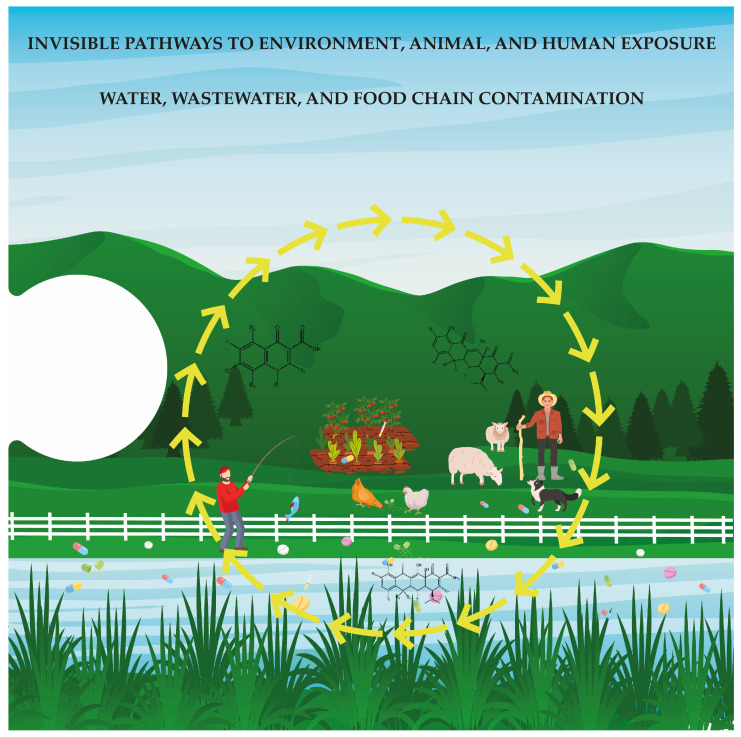
This final piece of the puzzle reveals how antibiotic residues and resistance genes spread beyond poultry farms. Antimicrobial resistance can affect soil, crops, animals, and humans through pathways such as contaminated litter, water, wastewater, and the food chain. These invisible routes highlight the interconnected risks and underscore the urgent need for a One Health approach to mitigate the global threat of antimicrobial resistance.

Antibiotics employed in poultry production function not only as growth promoters or disease control agents but also as persistent xenobiotics with extensive ecological impacts that extend beyond the farm. The chronic and fragmented nature of human exposure to these compounds and the antibiotic-resistant bacteria and genes they select for pose significant risks that remain under-recognized within current regulatory and surveillance frameworks. Furthermore, the concept that antibiotic concentration declines gradually from the point of application to the surrounding environment, resulting in sub-inhibitory levels, is critical for understanding the environmental consequences of antibiotic use, especially considering that these sub-inhibitory concentrations can facilitate genetic mutations, promote recombination events, and activate DNA repair mechanisms, ultimately contributing to the development of acquired antimicrobial resistance [17]. Therefore, these exposures are rarely acute or direct; rather, they accumulate silently through multiple pathways, including the ingestion of contaminated food, inhalation of bioaerosols, dermal contact with soil or water, and colonization by resistant microorganisms via environmental interfaces (Figure 3) [70,71,72]. Poultry environments serve as highly efficient hubs for the amplification and dissemination of AMR, particularly through the convergence of resistomes across animal, human, and environmental domains [70,73]. MDR *Enterococcus* species, for instance, have been isolated with high prevalence in both poultry and human populations, sharing clinically relevant resistance genes such as *vanA* and daptomycin resistance determinants, despite the latter not being used in veterinary settings [73]. Similarly, extended-spectrum β-lactamase-producing *E. coli* (ESBL-EC) strains, especially those harboring *bla*_CTX-M_ and *mcr-1*, have been detected in chickens, poultry environments, and humans, with a high genetic correlation suggesting transmission across compartments [71]. These findings support the hypothesis of resistome overlap and potential HGT between poultry-associated bacteria and the human microbiota [70,74].

Inhalation is a significantly under-recognized route of exposure. Airborne resistance genes, including *bla*_NDM_, *mcr-1*, *tet(X3)*, and *sul1*, have been identified in bioaerosols within and surrounding poultry facilities, extending up to 500 m from farm perimeters [72]. Individuals working in poultry environments and residents in proximity to these areas may experience chronic exposure to ARG-laden particles even in the absence of direct animal contact [70,75]. Bioaerosols have been demonstrated to contain multidrug-resistant *Staphylococcus aureus*, *Campylobacter* spp., and *Enterococcus* spp., frequently at concentrations exceeding the recommended occupational safety limits [70]. Consequently, the occupational setting emerges as a critical node in the transmission network, with poultry workers exhibiting higher colonization rates of resistant bacteria than unexposed populations [70]. Environmental reservoirs further complicate exposure dynamics. Poultry litter, soil, and untreated or inadequately treated wastewater have been shown to harbor high concentrations of ARGs and MDR pathogens, including linezolid-resistant *enterococci* [74,75,76]. These compartments facilitate the persistence, evolution, and transfer of resistance genes via MGEs such as plasmids and integrons, even in the absence of ongoing antibiotic pressure [74]. Such persistence poses a continuous low-level risk of exposure to populations through environmental contact and the food chain. Importantly, studies on antibiotic-free or organic production systems have revealed that antibiotic resistance does not necessarily disappear in the absence of antibiotics. Resistance genes have been detected in soil and poultry products in these systems, suggesting legacy contamination, environmental recirculation, or indirect selection mechanisms [76]. This highlights the inadequacy of binary distinctions between “antibiotic-free” and “conventional” as risk proxies and underscores the need for more nuanced risk assessments that consider cumulative and synergistic exposures. However, current policies often fail to account for the invisible and fragmented nature of human exposure to antibiotics and ARGs in poultry systems. Regulatory frameworks generally focus on maximum residue limits in food products or occupational exposure limits for specific pathogens without addressing the broader ecological and genetic risks of AMR dissemination [70,73].

Furthermore, the standardization of surveillance and exposure assessment methods remains limited, particularly with regard to airborne ARGs and the detection of subthreshold resistome signatures that become apparent only under gut-simulating conditions [74]. The complexity of these exposure pathways and the cumulative nature of their health effects necessitate a One Health approach that explicitly incorporates enduring, low-dose, and indirect exposures into AMR risk models. Such an approach must integrate environmental metagenomics, longitudinal epidemiology, and human microbiome studies to better characterize the burden of asymptomatic colonization, microbiota disruption, and potential clinical outcomes [70,71]. Without this integrative perspective, the contribution of poultry-derived xenobiotics to the global AMR crisis remains largely underestimated.

### 3.2. Resistome Convergence: From Farm to Clinic

Numerous metagenomic and metatranscriptomic investigations have revealed a significant overlap between ARGs identified in poultry environments and those present in human microbiomes or clinical pathogens. Commonly shared ARGs include those conferring resistance to tetracyclines (e.g., *tetA*, *tetB*, *tetM*, *tetO*, *tetQ*, *tetW*, *tetX*), macrolides-lincosamides-streptogramins (e.g., *ermB*), aminoglycosides (e.g., *aadA1*, *aph*(*3*′)-*Ia*, *aac*(*3*′)-VIa), and multidrug efflux pumps such as macA-macB and acrB-acrA-acrR [77,78,79,80,81,82]. These genes are extensively distributed across human, livestock, soil, and wastewater samples, constituting a shared resistome influenced by antimicrobial usage and environmental interactions.

The observed convergence is partially attributed to MGEs such as plasmids, integrons, transposons, and insertion sequences, which facilitate HGT. For example, the identification of a class 1 integron containing *aadA5-dfrA17* in chicken feces, identical to elements found in pathogenic *E. coli* and *Salmonella* strains, illustrates the circulation of clinically significant resistance cassettes between animal- and human-associated microbiota [77]. Similarly, IncI1 plasmids carrying *tetA*, *aadA1*, and *bla*_TEM-1B_ have been shown to be transferred from *E. coli* to *Salmonella* Heidelberg in poultry, providing direct evidence of in vivo gene exchange [11]. Active MGEs, particularly transposases such as IS91, were more prevalent in poultry samples, further implicating the chicken gut as a hotspot for ARG mobilization [78].

Although strain-level tracking is not consistently available, certain studies have identified sequence types associated with human infections, such as *E. coli* ST69, which is prevalent in poultry environments [11]. The presence of the colistin resistance gene *mcr-1* in chicken and pig samples, but not in humans, suggests that ARGs may be indirectly transmitted to humans through intermediate reservoirs, such as food animals [78,81]. Furthermore, genes such as *ermB* and *tetQ*, which are present in both hospital patients and poultry, are often located on conjugative elements and have been demonstrated to facilitate transfer between commensal and pathogenic bacteria [81].

The principal pathways for the transmission of ARGs from poultry to humans include foodborne exposure, contaminated water, contact with manure-amended soils, and occupational exposure. ARGs identified in animal gut microbiomes have been detected in meat products and wastewater effluents, implicating the food chain and aquatic environments as important conduits for resistome transfer [77,79,82]. The application of poultry manure as fertilizer introduces substantial loads of ARGs and MGEs into soil and crops, thereby increasing the risk of transferring resistance to fresh produce [82]. The presence of shared ARGs and microbial taxa in humans residing near intensive animal farming regions further supports the hypothesis of local environmental spillover [81].

Ecological and chemical co-selective pressures, such as the presence of heavy metals in manure, may independently sustain ARGs by facilitating the persistence of co-located resistance genes and MGEs [77,82]. Furthermore, ARGs have been identified in genera typically regarded as beneficial, such as *Lactobacillus*, which raises concerns regarding their dissemination through probiotic or commensal bacterial pathways [79,81].

These findings demonstrate a strong interconnection between poultry farming and clinical antimicrobial resistance, with the convergence of resistomes serving as a quantifiable outcome of the shared selective pressures and genetic exchange. The clinical implications are substantial: the enrichment of last-resort resistance genes, such as *mcr-1*, in food animals increases the risk of therapeutic failure in human medicine, whereas shared resistance profiles complicate infection control and antibiotic stewardship. Despite these advancements, strain-level tracking of ARG-carrying pathogens across the food–animal–human continuum remains limited. Future studies should prioritize whole-genome sequencing and functional genomics to directly link environmental resistomes to human clinical isolates. Additionally, quantifying the relative contributions of different transmission routes, particularly the roles of food, water, and occupational exposure, is crucial for guiding effective intervention strategies. Collectively, these findings underscore the urgent need for integrated One Health surveillance systems and policy frameworks that acknowledge the bidirectional flow of resistance genes between farms and clinics. The surveillance of mobile and transcriptionally active ARGs, particularly in poultry, is essential for assessing real-time risks and preventing the spread of resistance across ecological boundaries [83,84,85,86].

### 3.3. One Health: Ethical Imperative and Context-Specific Implementation

**Figure 4:**
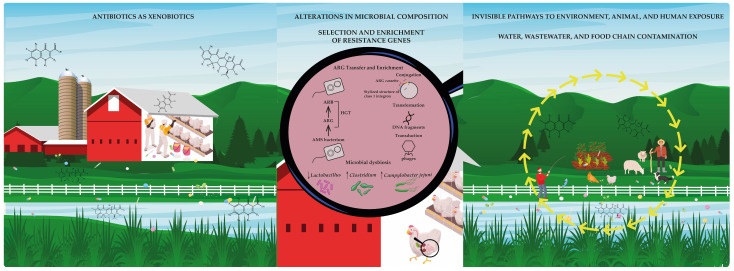
The complete puzzle: a systemic view of antimicrobial resistance. When all pieces come together, they reveal a comprehensive and interconnected picture of antimicrobial resistance. This visual synthesis highlights the links between antibiotic use in poultry farming, the disruption of microbial ecology, environmental dissemination, and human exposure. It underscores the urgent need for an ethical and integrated response grounded in the One Health framework and guided by the precautionary principle.

The proliferation of AMR across human, animal, and environmental systems underscores the critical need for a One Health approach to public health protection. Poultry and other food-producing animals frequently act as reservoirs for MDR pathogens, such as *C. jejuni* and *E. coli*, thereby facilitating the transmission of resistance genes and virulence factors through food, water, and occupational contact [87,88,89]. Globally, shared resistance profiles and genetic elements across clinical, animal, and environmental isolates have been documented, reflecting the convergence of selective pressures driven by the extensive use of antibiotics in agriculture [86,90,91]. The integration of antibiotics into animal production systems, which often lacks professional oversight, has resulted in ecological saturation with resistance determinants. In many low- and middle-income countries, unregulated or prophylactic antibiotic use persists, with drugs administered not only for disease control but also for growth promotion [92,93,94]. Poultry litter and untreated wastewater from farms are significant sources of environmental contamination that contribute to the dispersal of resistant bacteria and antibiotic residues across soil, water, and food crops [95,96]. These residues function as xenobiotics, environmentally persistent, biologically active compounds capable of exerting selective pressure on microbial communities and altering the resistome composition (Figure 4) [89,95].

Genomic analyses revealed clonal relationships and horizontal gene transfer between the human and animal isolates, further substantiating the interconnected dynamics of resistance dissemination [86,91]. Resistance genes, such as *bla*_CTX-M_, *tet*, *cme*, and *van*, have been identified across multiple domains, underscoring the ecological entrenchment of AMR within microbial networks [73,87,88]. The presence of mobile genetic elements enhances the persistence and mobility of resistance traits, thereby posing a threat to the efficacy of critical antibiotics in human medicine [89,91,97]. Although a few studies explicitly invoke the precautionary principle, many implicitly reflect on its rationale. The environmental dissemination of resistance genes, their detection in food and water, and the lack of regulatory controls underscore the ethical imperative for preventive action [92,95,96]. The precautionary perspective frames AMR not only as a scientific challenge but also as a matter of intergenerational justice, where continued inaction compromises future treatment options and burdens vulnerable populations with avoidable health risks [87,97]. Therefore, the One Health strategy is not merely a technical recommendation but an ethical necessity. It provides a framework for coordinated surveillance, prudent antimicrobial use, and cross-sectoral collaboration, as evidenced by the national action plans, monitoring systems, and stewardship programs emerging across Asia, Africa, and Europe [73,94,96]. Preventive strategies, including alternatives to antibiotics, environmental hygiene, regulatory enforcement, and stakeholder education, are essential to disrupt the transmission of resistance at their source [92,93,98]. Ultimately, addressing AMR as a xenobiotic-driven phenomenon requires an integrated ethically grounded response. The resilience and adaptability of resistance genes in natural and human-modified ecosystems illustrates the utility of isolated interventions. Only through the comprehensive implementation of the One Health principle, anchored in precaution and stewardship, can invisible pathways of human antibiotic exposure be effectively disrupted.

Implementing a One Health approach to AMR in poultry production encounters substantial structural and behavioral challenges, particularly in low- and middle-income countries. Inappropriate practices driven by insufficient knowledge of antimicrobial stewardship are prevalent among poultry farmers, who frequently use antibiotics indiscriminately for growth promotion and disease prevention without veterinary oversight. This misuse is exacerbated by weak regulatory frameworks and limited enforcement capacity, which fail to curb the overuse and misuse of antibiotics in poultry systems [99,100]. Further complicating the issue, diagnostic capabilities are often inadequate, leading to empirical prescription by veterinarians without culture and susceptibility testing, while economic constraints drive farmers to favor low-cost antibiotics over more sustainable but costlier alternatives [101]. Environmental contamination adds another layer of complexity, as improper disposal of poultry waste containing antimicrobial residues facilitates the dissemination of resistance genes into the soil and water, creating reservoirs of resistance beyond farm boundaries. Close human–animal contact and poor biosecurity measures further enable the transmission of resistant bacteria among poultry, farm workers, and the surrounding community, thereby threatening public health [100]. These challenges are compounded by limited awareness of AMR risks among stakeholders as well as insufficient surveillance systems to monitor antibiotic use and resistance patterns in poultry production, making coordinated action difficult to design and implement [101]. Overcoming these barriers necessitates a coordinated, multi-sectoral strategy embedded within the One Health framework. Education and training initiatives targeting farmers, veterinarians, and consumers are essential to promote responsible antibiotic use and raise awareness about AMR risks. Strengthening regulatory and enforcement mechanisms along with harmonizing policies across regions can help curb inappropriate practices. Investment in affordable diagnostic tools and alternative interventions, such as vaccines, probiotics, and enhanced biosecurity, can reduce dependence on antibiotics while maintaining productivity. Economic incentives and funding support are also critical to enable farmers to adopt antimicrobial stewardship practices without compromising their livelihoods [99,101]. Robust surveillance and monitoring systems are needed to track antibiotic use and resistance trends, and improved waste management practices can mitigate environmental contamination. Finally, fostering cross-sectoral collaboration among the human, animal, and environmental health sectors ensures that AMR is addressed holistically, in line with the ethical imperative of protecting both public health and ecological integrity [100,101]. Beyond poultry, antimicrobial resistance risks and mitigation strategies vary considerably across different geographical regions and production systems. This variability is shaped by diverse regulatory frameworks, agricultural practices, environmental conditions, and socioeconomic contexts, all of which must be accounted for when designing effective context-specific interventions. For instance, in Nigeria, urban and rural areas encounter distinct challenges owing to disparities in access to veterinary services, infrastructure, and education, emphasizing the need for mitigation efforts tailored to local realities [99]. Similarly, regional disparities documented across the northwest, southeast, and north–central zones indicate that AMR prevalence and drivers are not homogeneous, underscoring the importance of regionally adapted policies [99]. Production systems significantly influence AMR risk profiles. Intensive poultry operations, characterized by high animal densities and industrialized practices, often exacerbate the dissemination of resistant bacteria owing to increased antibiotic usage and enhanced transmission opportunities within flocks. Conversely, extensive backyard systems, which are more prevalent in rural and lower-income regions, present distinct risks associated with limited veterinary oversight and inadequate biosecurity measures [100,101]. Environmental factors such as climate variability, water quality, and waste management practices also affect AMR dynamics by influencing the persistence and dissemination of resistance genes within ecosystems [100]. Addressing these multifaceted, context-dependent challenges necessitates mitigation strategies that are both adaptable and informed by the local conditions. A One Health approach tailored to specific regional and production system contexts rather than being imposed as a uniform model is crucial. This includes customizing education and stakeholder engagement programs to local languages, cultural norms, and knowledge levels, to ensure that farmers and veterinarians can adopt responsible antibiotic practices [99]. Surveillance and diagnostic systems must be designed to align with regional capacities, potentially utilizing mobile- and cloud-based technologies to address infrastructure limitations [101]. Similarly, economic incentives promoting stewardship and alternatives to antibiotics, such as vaccination, probiotics, and enhanced biosecurity, must reflect the financial realities of the target communities [100]. Experiences from high-income countries demonstrate that well-designed, system-specific interventions can be successful and offer valuable lessons for adaptation in low-income and middle-income settings. For instance, Denmark’s “Yellow Card” initiative launched in 2010 exemplifies how targeted policies can achieve substantial reductions in antimicrobial use without compromising productivity. By setting threshold limits and enforcing compliance, Denmark has reduced antibiotic consumption in livestock by 27% while maintaining animal health and efficiency [102,103]. Similarly, the Netherlands implemented a comprehensive, multi-pronged strategy that combined mandatory reduction targets, improved diagnostics, a ban on growth-promoting antibiotics, and extensive education, achieving a remarkable 64% reduction in veterinary antibiotic use between 2007 and 2020 [104]. At a broader level, the European Union established harmonized regulations banning antibiotics for growth promotion as early as 2006, complemented by standardized monitoring and stewardship guidelines through the European Medicines Agency (EMA) and European Food Safety Authority (EFSA) [105]. Advanced surveillance platforms, such as Denmark’s VetStat, have been critical in tracking antimicrobial use and resistance in real time, enabling evidence-based policy adjustments, and maintaining the effectiveness of stewardship interventions [106]. Countries have also explored alternative strategies to reduce their reliance on antibiotics. In France, replacing in-feed antibiotics with essential oils and organic acids significantly reduced colistin-resistant *E. coli* on poultry farms [107]. Germany and Switzerland tested phytogenic feed additives such as garlic and oregano extracts to maintain animal health while minimizing infections. Similarly, China has successfully implemented probiotic supplementation in swine production to reduce reliance on tetracyclines and macrolides, curbing the spread of resistance genes [105,108]. Promising initiatives have emerged in low- and middle-income countries. For instance, the FAO ATLASS (Assessment Tool for Laboratories and Antimicrobial Resistance Surveillance Systems (ATLASS)) has enhanced local capacities for the surveillance and management of AMR, promoting cross-border collaboration and facilitating the development of localized, sustainable strategies that contribute to global efforts [109]. These examples collectively highlight the necessity of adapting mitigation strategies to the specific regulatory, economic, and cultural contexts of each region and its production system. By learning from successful models and tailoring interventions accordingly, it is possible to align local practices with the broader One Health framework, ensuring that AMR risks are effectively addressed while preserving food security, animal welfare, and environmental health.

## 4. Conclusions

From a medical perspective, antibiotics were made to act in the bodies of sick people or animals, targeting single pathogens; however, the use of these bio-powered substances (treatment, prophylaxis, metaphylaxis, and growth promotion) is an ecological event that drives bacterial evolution in bodies and far beyond them, disrupting biological systems and driving AMR. Their effects are concentration-dependent, and sub-inhibitory concentrations are especially problematic, as they can promote the development of acquired resistance. Moreover, the specific outcomes vary depending on the ecological context.

In the gut microbiome, antibiotic administration can reduce microbial diversity, alter taxonomic composition, deplete protective genera, increase the abundance of resistance genes, and disrupt host metabolic functions. Antibiotic exposure also leads to selective pressure that enables horizontal gene transfer and the co-selection of resistance to antibiotics that are not directly administered.

Furthermore, in airborne microbism, which often originates from fecal matter, antibiotic use is associated with lower diversity and increased resistance. Bioaerosols can disperse into the surrounding environment, which is particularly concerning in animal production industries where routine disinfection practices fail to eliminate resistance strains in the air across production cycles. This presents a risk not only to the environment but also to workers and nearby communities. Water systems serve as reservoirs and transmission routes of resistance, even in farms where antibiotics have not been actively used. The existence of biofilms that allow the survival of MDR bacteria greatly contributes to this effect. In such cases, addressing AMR requires not only better antibiotic stewardship but also infrastructural improvement. Poultry litter, comprising bedding, feces, feathers, and feed residues, acts as a long-term environmental reservoir for resistance. Its application as a fertilizer, especially if untreated, facilitates the persistence of MDR microorganisms in the soil for extended periods, representing a public health risk.

Major transmission routes of ARG from poultry to humans include foodborne exposure, contaminated water, contact with manure-amended soil, and occupational exposure. Although studies are scarce, the same strains have been documented in clinics and poultry production, underscoring the urgent need for comprehensive AMR surveillance. Ultimately, these findings emphasize the need for integrated, cross-sectoral approaches grounded in the One Health framework, recognizing the interdependence between human, animal, and environmental health, particularly in AMR contexts.

## Figures and Tables

**Table 1:** Classes of antibiotics used in poultry production, their mechanisms of action, intended purposes, and unintended consequences as xenobiotics. Adapted and expanded from Islam et al. (2024) [26] and Bacanlı (2024) [27]. These antibiotics, though originally of microbial, semi-synthetic, or synthetic origin, behave as xenobiotics when administered to poultry, persisting in animal tissues, manure, soil, water, and food products, and contributing to antimicrobial resistance and ecological disruption.

Class/Example	Origin	Uses in Poultry Production	Justification as Xenobiotic *
Tetracyclines (e.g., oxytetracycline)	Naturally derived (*Streptomyces*) and semi-synthetic derivatives	Therapeutic, prophylactic, growth promotion	Foreign to poultry microbiota and ecosystems; excreted into environment, exerting selective pressure
Macrolides (e.g., tylosin)	Naturally derived (*Streptomyces*)	Therapeutic and prophylactic	Disruptive to microbial communities and detected as residues in soil and water
Beta-lactams (e.g., amoxicillin)	Naturally derived (*Penicillium*) and semi-synthetic	Therapeutic	Not produced by poultry; persists in manure; selects for resistance genes
Sulfonamides (e.g., sulfamethazine)	Fully synthetic	Therapeutic and prophylactic	Synthetic compounds, foreign to all biological systems
Fluoroquinolones (e.g., enrofloxacin)	Fully synthetic	Therapeutic	Fully synthetic, foreign to ecosystems; strong ecological impact
Aminoglycosides (e.g., gentamicin)	Naturally derived (*Micromonospora*) and semi-synthetic	Therapeutic	Non-host compounds, environmentally persistent
Polypeptides (e.g., colistin)	Naturally derived (Bacillus, Paenibacillus)	Growth promotion, therapeutic	Alters gut flora and environmental microbiota despite natural origin

* Xenobiotics refer to compounds not naturally produced nor expected to be present in an organism, which includes foreign chemicals, toxins, or pharmaceuticals such as antibiotics.

## Data Availability

No new data were created or analyzed in this study. Data sharing is not applicable to this article.
